# Silencing of Poly(ADP-Ribose) Polymerase-2 Induces Mitochondrial Reactive Species Production and Mitochondrial Fragmentation

**DOI:** 10.3390/cells10061387

**Published:** 2021-06-04

**Authors:** Laura Jankó, Tünde Kovács, Miklós Laczik, Zsanett Sári, Gyula Ujlaki, Gréta Kis, Ibolya Horváth, Miklós Antal, László Vígh, Bálint L. Bálint, Karen Uray, Péter Bai

**Affiliations:** 1 Department of Medical Chemistry, Faculty of Medicine, University of Debrecen, H-4032 Debrecen, Hungary; janko.laura@med.unideb.hu (L.J.); kovacs.tunde@med.unideb.hu (T.K.); sari.zsanett@med.unideb.hu (Z.S.); ujlaki.gyula@med.unideb.hu (G.U.); karen.uray@med.unideb.hu (K.U.); 2 Department of Biochemistry and Molecular Biology, Faculty of Medicine, University of Debrecen, H-4032 Debrecen, Hungary; laczik.miklos@med.unideb.hu (M.L.); lbalint@med.unideb.hu (B.L.B.); 3 Department of Anatomy, Histology and Embryology, Faculty of Medicine, University of Debrecen, H-4032 Debrecen, Hungary; greta@anat.med.unideb.hu (G.K.); antal@anat.med.unideb.hu (M.A.); 4 Biological Research Center of the Hungarian Academy of Sciences, H-6726 Szeged, Hungary; hibi@lipidart.com (I.H.); vigh@lipidart.com (L.V.); 5 MTA-DE Neuroscience Research Group, H-4032 Debrecen, Hungary; 6 MTA-DE Lendület Laboratory of Cellular Metabolism, H-4032 Debrecen, Hungary; 7 Research Center for Molecular Medicine, Faculty of Medicine, University of Debrecen, H-4032 Debrecen, Hungary

**Keywords:** PARP2, ARTD2, oxidative stress, mitochondrial biogenesis, skeletal muscle, mitochondrial fragmentation, mitochondrial morphology

## Abstract

PARP2 is a DNA repair protein. The deletion of PARP2 induces mitochondrial biogenesis and mitochondrial activity by increasing NAD^+^ levels and inducing SIRT1 activity. We show that the silencing of PARP2 causes mitochondrial fragmentation in myoblasts. We assessed multiple pathways that can lead to mitochondrial fragmentation and ruled out the involvement of mitophagy, the fusion–fission machinery, SIRT1, and mitochondrial unfolded protein response. Nevertheless, mitochondrial fragmentation was reversed by treatment with strong reductants, such as reduced glutathione (GSH), N-acetyl-cysteine (NAC), and a mitochondria-specific antioxidant MitoTEMPO. The effect of MitoTEMPO on mitochondrial morphology indicates the production of reactive oxygen species of mitochondrial origin. Elimination of reactive oxygen species reversed mitochondrial fragmentation in PARP2-silenced cells.

## 1. Introduction

Optimal mitochondrial biogenesis requires the coordinated expression of nuclear and mitochondrial genes. Most mitochondrial genes are coded in the nucleus. There is a dominant flow of information and material from the nucleus and the cytosol to the mitochondria, termed anterograde signaling [1]. The appropriate flux of protein from the cytosol to the mitochondria requires the coordinated regulation of nuclear transcription by a large set of nuclear transcription factors [2]. This web of transcription factors responds to changes in nutrient levels (amino acids, oxygen, NAD^+^, etc.) and cellular energy charge. Therefore, the transcriptional programs can accommodate the actual nutrient and energy charge of cells [3,4,5,6]. The disruption of nutrient sensing and mitonuclear proteostasis plays a pathogenic role in metabolic and neoplastic diseases [7,8,9].

An increase in the mitochondrial content of cells is termed mitochondrial biogenesis [2,10]. Mitochondria undergo constant quality control [10,11,12,13,14,15], in which, among others, mitophagy [16,17,18] and the mitochondrial unfolded protein response (mtUPR) [19,20] play a major role. Mitochondrial activity and mitochondrial quality control modulate the structure of the cellular mitochondrial network. Higher mitochondrial output usually accompanies fusion of the mitochondrial network, while removal of damaged mitochondrial components is associated with the fragmentation of the mitochondrial network [12,21,22,23].

poly(ADP-ribose) polymerases constitute a large protein family (PARP/ARTD1-17). Members of the PARP/ARTD family share the PARP catalytic domain that can cleave NAD^+^ to nicotinamide and ADP-ribose, subsequently using ADP-ribose as building blocks to build large poly(ADP-ribose) polymers (PAR) [24,25]. PARP2 accounts for around 15% of total cellular PARP activity, as a function of the model system used [26,27,28,29]. PARP2 can be activated by DNA strand breaks, irregular DNA forms [29,30,31], lipid species [32,33], and signal transduction pathways [34,35,36]. PARP2 is primarily localized to the nucleus [29,37] and is highly expressed in the testes [29], thymus, central nervous system, liver, cortex of the kidney, stomach, and intestinal epithelia [26]. PARP2 was first described as a DNA remodeling and repair enzyme [26,29,38,39,40] with a role in tumor biology [41,42,43,44,45]. Since then, other physiological and pathophysiological roles were identified for PARP2 in spermiogenesis [40,46], immune function [47,48,49,50,51], oxidative injury [27,52,53], metabolic and mitochondrial regulation [33,54,55,56,57], autophagy [58], cancer cachexia [59,60], and transcription [33,48,49,50,54,56,61] (we refer the readers to thorough reviews [35,36]). Importantly, the inhibition of PARP2 is responsible for many of the side effects of clinical PARP inhibitors [49,50].

PARP2 is involved in mitochondrial and metabolic regulation. The interactions are multipronged; nevertheless, from the perspective of our study, two biological processes are vital for understanding the interplay between SIRT1 and PARP2 and the involvement of PARP2 in autophagy. SIRT1 is a NAD^+^-dependent protein deacetylase [62] that can deacetylate and, hence, induce key mitochondrial transcription factors, such as the peroxisome proliferator-activated receptor cofactor-1α (PGC1α) or the forkhead box protein O1 (FOXO1) [63,64,65,66,67]. PARP2 can induce SIRT1 expression and activity that consequently leads to mitochondrial biogenesis [27,55,57]. Furthermore, we recently showed that the genetic deletion or silencing of PARP2 blocks autophagy [58], which can influence mitochondrial morphology and mitochondrial quality control. In that study, our aim was to assess how PARP2 affects mitochondrial morphology.

## 2. Materials and Methods

### 2.1. Chemicals

All chemicals, glutathione (GSH), N-acetyl-L-cysteine (NAC), and MitoTEMPO, were from Sigma-Aldrich (St. Louis, MO, USA). GSH (cat. no. G4251) and NAC (cat. no. A7250) antioxidants were used at a final concentration of 5 mM for 48 h. A mitochondria-targeted antioxidant MitoTEMPO (cat. no. SML0737) was used at a concentration of 10 µM for 48 h.

### 2.2. Cell Culture

PARP2-silenced C2C12 cells were described in [55]. C2C12 cells were cultured in DMEM (cat. no. D6429, Sigma-Aldrich, 4500 mg/L glucose) containing 10% FBS, 1% penicillin/streptomycin, and 2 mM L-glutamine at 37 °C with 5% CO_2_.

PARP2-silenced HepG2 cells were described in [54]. HepG2 cells were cultured in DMEM (cat. no. D5546, Sigma-Aldrich, 1000 mg/L glucose) containing 10% FBS, 1% penicillin/streptomycin, and 2 mM L-glutamine at 37 °C with 5% CO_2_.

**Figure 1:**
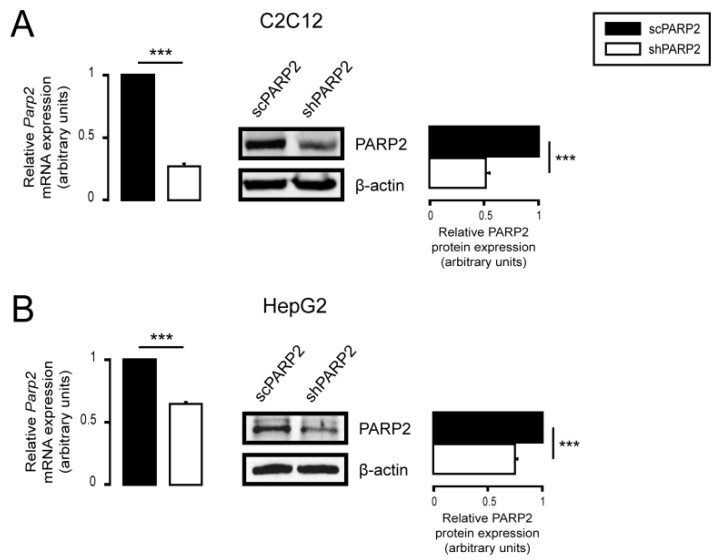
Silencing of PARP2 in C2C12 myoblasts and HepG2 cells. (**A**) A total of 200,000 scPARP2 or shPARP2 C2C12 cells were seeded into 6-well plates, and the expression of PARP2 was determined by RT-qPCR (*n* = 3) and Western blotting (*n* = 3). (**B**) A total of 250,000 scPARP2 or shPARP2 HepG2 cells were seeded into 6-well plates, and the expression of PARP2 was determined by RT-qPCR (*n* = 3) and Western blotting (*n* = 3). Representative Western blot images are presented. Numerical values are presented as the average ± SEM. Statistical significance was determined using paired, two-tailed Student’s *t*-test. *** Statistically significant differences between scPARP2 and shPARP2 C2C12 cells or scPARP2 and shPARP2 HepG2 cells at *p* < 0.001.

PARP2 was silenced by specific shRNA in both cell lines and maintained over extended periods by selection with 2.5 µg/mL of puromycin for C2C12 cells and with 0.25 µg/mL of puromycin for HepG2 cells. Cells that harbor the plasmid expressing the small hairpin RNA against PARP2 (i.e., PARP2 silenced) are referred to as shPARP2, while cells that harbor the plasmid expressing a small hairpin RNA that has no target in human or murine cells (controls) are referred to as scPARP2. Both cell lines were established earlier [54,55], and the effectiveness of silencing was verified before experimentation (Figure 1A,B).

### 2.3. Transient Transfection

Silencer Select siRNAs were purchased from Thermo Fisher Scientific (Walthan, MA, USA). SiRNAs targeting PARP2 (cat. no. 4390771, ID: s62056 as #1, s62057 as #2, s62058 as #3), SIRT1 (cat. no. 4390771, ID: s96766), PARP1 (cat. no. 4390771, ID: s62053 as #1, s62054 as #2, s62055 as #3), PARP3 (cat. no. 4390771, ID: s108205 as #1, s108206 as #2, s108207 as #3), and negative control (cat. no. 4390843) were used. Cells were plated in 24-well plates and transfected with siRNA at a final concentration of 30 nM using Lipofectamine RNAiMax reagent (cat. no. 13778075, Invitrogen, Carlsbad, CA, USA). Cells were assayed 48 h post-transfection.

### 2.4. In Vitro Cell Proliferation Assay (SRB Assay)

Cellular proliferation was determined using Sulphorhodamine B (SRB) assay, as described in [68].

### 2.5. Detection of Cell Death

To evaluate changes in apoptotic and necrotic cell death, an FITC Annexin V/Dead Cell Apoptosis Kit (cat. no. V13242, Invitrogen) was used according to the manufacturer’s instructions. Cells were seeded into 6-well plates and treated with the different chemicals, as stated. Then, cells were collected and stained with 5 µL of FITC annexin V and 100 µg/mL of PI for 15 min at room temperature. Cells were analyzed by flow cytometry (FACS Calibur, Becton Dickinson Biosciences, San Jose, CA, USA), and data were analyzed using BD CellQuest Pro software v5.2 (Becton Dickinson Biosciences).

### 2.6. Determination of Cellular ATP Level

Cells were seeded into 6-well plates and treated, as indicated. ATP levels were determined using an ATP Assay Kit (cat. no. MAK190, Sigma-Aldrich, St. Louis, MO, USA). ATP concentration was measured in 96-well black plates using a fluorimeter (Spark 20M, Tecan Life Sciences, Männedorf, Switzerland). ATP levels were normalized to protein content, and normalized readings are presented.

### 2.7. MitoTracker Red Staining

Cells grown on glass coverslips were treated with the specified chemicals, as indicated, or transfected with the indicated siRNAs (see Figure legend). Mitochondria were stained with MitoTracker Red, as described in [69]. Confocal images were acquired with a Leica TCS SP8 confocal microscope (Leica, Wetzlar, Germany) and LAS X software v3.5.5.19976 (Leica). Processed images were analyzed using ImageJ v1.44 software with Mito-Morphology Macro [70], yielding the mitochondrial number, content, circularity, and form factor. Form factor is derived from the area-to-perimeter ratio [71]; hence its decrease would signify fragmentation. Circularity increases if the shape of an object is closer to a circle; hence, increased circularity suggests that the mitochondria are not elongated, which is a feature of a disassembled mitochondrial network. Perimeter is a similar term; a decrease in perimeter indicates smaller mitochondria. For co-localization analysis and the assessment of the Pearson correlation coefficient, ImageJ software with EzColocalization plug-in was used [72].

### 2.8. Immunofluorescence

Immunofluorescence was described in [58]. Antibodies used in immunofluorescence are listed in Table 1.

### 2.9. Total RNA Preparation and Reverse Transcription–Quantitative PCR (RT-qPCR)

Total RNA preparation, reverse transcription, and qPCR were performed, as described in [58]. Expression was normalized to the geometric mean of murine 36B4 and cyclophilin values. Primers are listed in Table 2.

### 2.10. SDS-PAGE and Western Blotting

SDS-PAGE and Western blotting were described in [58]. Primary and secondary antibodies are listed in Table 3. Bands were quantified by densitometry using ImageJ software v1.44 [73], and densitometry data were analyzed by statistical methods.

### 2.11. Determination of Lipid Peroxidation (TBARS Assay)

Lipid peroxidation was assessed by determining the production rate of thiobarbituric acid-reactive substances (TBARS) similar to [74].

### 2.12. Determination of the Oxygen Consumption Rate (OCR)

The oxygen consumption rate was determined using the XF96 Flux Analyzer (Agilent Technologies, Santa Clara, CA, USA). Cells were seeded into 96-well assay plates and treated with the indicated concentration of different chemicals for 48 h (see Figure legend). After recording the baseline, the OCR was recorded every 5 min, and 50 µM etomoxir (cat. no. E1905, Sigma-Aldrich) was used for determining the fatty acid oxidation. Data were normalized to protein content, and normalized readings are displayed.

### 2.13. Measurement of Superoxide Production (DHE Staining)

Superoxide production was measured using dihydroethidium (DHE) staining. Cells were seeded into 6-well plates for 1 day. Cells were stained with 2.5 µM DHE (cat. no. D7008, Sigma-Aldrich) for 30 min, and fluorescence was analyzed by flow cytometry (FACSCalibur, Becton Dickinson Biosciences, San Jose, CA, USA). For data evaluation, BD CellQuest Pro software v5.2 (Becton Dickinson Biosciences) was used.

### 2.14. Electron Microscopy

Data from a previous study [58] were re-analyzed. All relevant methodology can be found in [58].

### 2.15. Statistical Analysis

Statistical analyses were performed using GraphPad Prism v8 software (GraphPad Software, San Diego, CA, USA). The number of repetitions for each experiment is indicated in the figure legends. All groups were checked for normal distribution. For comparing two groups, two-sided Student’s t-tests were applied. For multiple comparisons, one-way ANOVA was conducted, followed by Tukey’s post hoc test (to compare all possible combinations) or Dunnett’s post hoc test (to compare data to one selected group). The levels of significance are indicated in figure captions; *n* denotes the number of biological replicates.

## 3. Results

### 3.1. Silencing of PARP2 Leads to Fragmented Mitochondria

**Figure 2:**
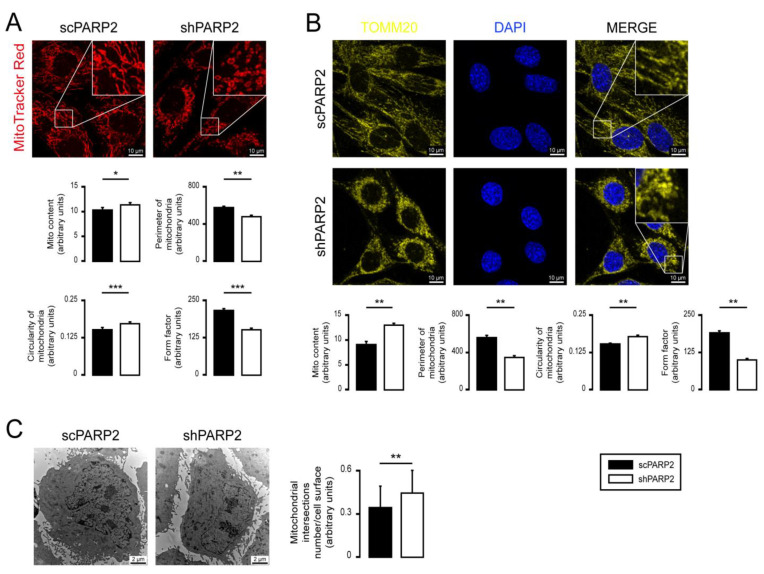
Silencing of PARP2 leads to mitochondrial fragmentation. (**A**,**B**) A total of 70,000 scPARP2 or shPARP2 C2C12 cells were seeded into 24-well plates on glass coverslips, and the cells were labeled with MitoTracker Red (*n* = 3) (**A**) or TOMM20 antibody (**B**). The nuclei were visualized using DAPI (*n* = 3). Mitochondrial morphology was analyzed using ImageJ software with Mito-Morphology Macro (measured cells: 20/20). (**C**) Osmiated scPARP2 and shPARP2 C2C12 cells were analyzed by electron microscopy (*n* = 1; 45 cells were assessed from each group). Mitochondrial numbers were determined using ImageJ software, and mitochondrial intersections number/cell surface were plotted. Representative immunofluorescence, electron microscopic, and Western blot images are presented in the figure. Numeric values are presented as the average ± SEM in (**A**,**B**) and as the average ± SD in (**C**). Statistical significance was determined using paired, two-tailed Student’s *t*-test in (**A**–**C**). *, **, and *** Statistically significant differences between the indicated groups at *p* < 0.05, *p* < 0.01, and *p* < 0.001, respectively.

**Figure 3:**
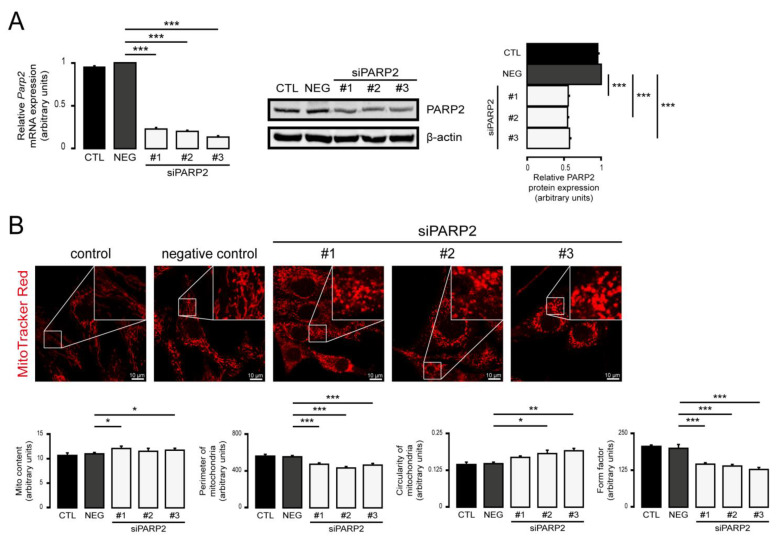
Acute silencing of PARP2 leads to mitochondrial fragmentation. (**A**,**B**) A total of 30,000 C2C12 cells were seeded into 24-well plates, and PARP2 was transiently silenced using three types of siRNAs (*n* = 3). Cells were assessed 48 h post-transfection, then the expression of PARP2 was determined using RT-qPCR (*n* = 3) and Western blotting (*n* = 3) (**A**). In the same cells, mitochondrial structure was investigated using MitoTracker Red staining (*n* = 3) (**B**). Mitochondrial morphology was analyzed using ImageJ software with Mito-Morphology Macro (measured cells: 20 from each group). Representative Western blot and immunofluorescence images are presented. Numerical values are presented as the average ± SEM. Statistical significance was determined using ANOVA followed by Dunnett’s post hoc test. *, **, and *** Statistically significant differences between the negative control and transiently transfected samples at *p* < 0.05, *p* < 0.01, and *p* < 0.001, respectively.

We assessed the mitochondrial morphology in scPARP2 and shPARP2 C2C12 myoblasts. Silencing of PARP2 induced the mitochondrial content in cells (a readout called “Mito Content”), as visualized by Mitotracker Red (Figure 2A) and TOMM20 immunostaining (Figure 2B), in good agreement with previous observations [27,55,57]. In addition, the silencing of PARP2 resulted in fragmentation of the mitochondrial network, marked by increased circularity and decreased individual mitochondria perimeters and form factors (Figure 2A,B). We re-analyzed electron microscopy sections from a previous study [58] and found that the number of mitochondrial cross sections increase in cells, which indicates the fragmentation of mitochondria (Figure 2C). Acute silencing of PARP2 by siRNA in C2C12 cells led to the induction of mitochondrial content and fragmentation of the mitochondrial network, similar to the findings in the established cell line (Figure 3A–C). Pharmacological inhibition of PARP1 by olaparib or nicotinamide was shown to induce mitochondrial fragmentation [20], similar to boosting in cellular NAD^+^ levels [75,76,77,78,79,80] that aligns well with our observations.

### 3.2. SIRT1 Activation, Mitophagy, mtUPR, and the Deregulation of the Mitochondrial Fusion–Fission System Are Not Involved in Mitochondrial Fragmentation in PARP2-Silenced Cells

**Figure 4:**
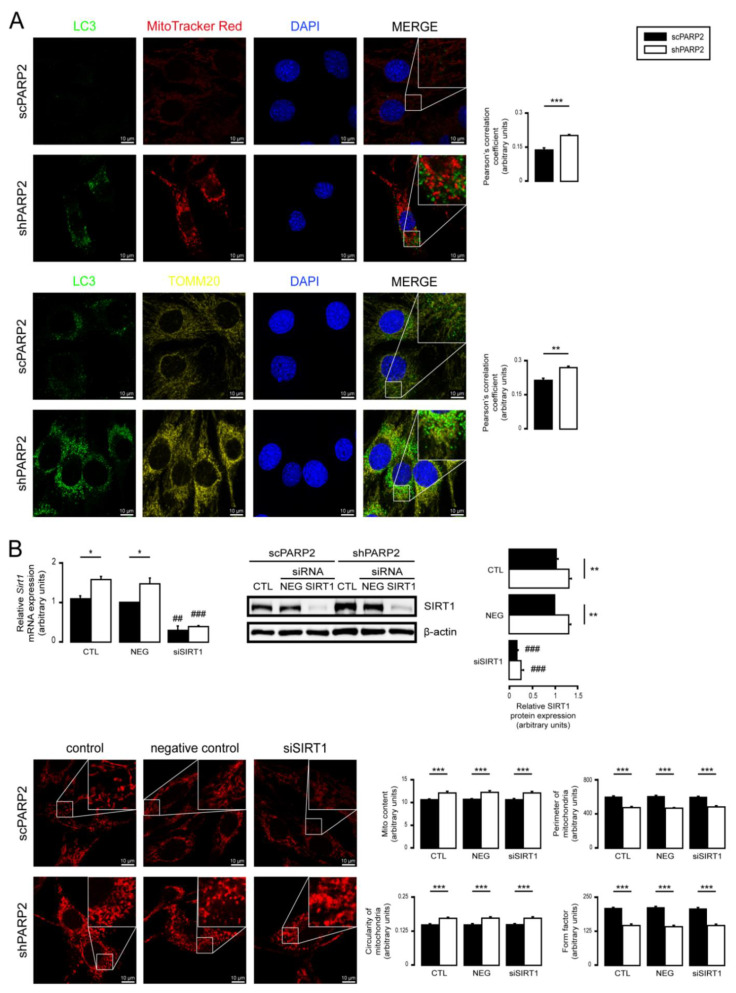
Mitochondrial fragmentation in shPARP2 C2C12 is not due to mitophagy or SIRT1 activation. (**A**) A total of 70,000 scPARP2 or shPARP2 C2C12 cells were seeded into 24-well plates on glass coverslips, and LC3-MitoTracker Red–DAPI or LC3-TOMM20–DAPI co-immunofluorescence was performed (*n* = 3). Co-localization analysis was performed using ImageJ software with the EzColocalization plug-in (measured cells: 100/100). (**B**) A total of 30,000 scPARP2 or shPARP2 C2C12 cells were seeded into 24-well plates, and SIRT1 was transiently silenced. After transfection, the expression of SIRT1 was determined using RT-qPCR (*n* = 3) and Western blotting (*n* = 3). The mitochondrial structure was investigated using MitoTracker Red staining (*n* = 3). Mitochondrial morphology was analyzed using ImageJ software with Mito-Morphology Macro (measured cells: 20 from each group). Representative immunofluorescence and Western blot images are presented in the figure. Numerical values are presented as the average ± SEM. Statistical significance was determined using paired, two-tailed Student’s *t*-test in (**A**), while in (**B**), ANOVA followed by Tukey’s post hoc test was used. *, **, and *** Statistically significant differences between the scPARP2 and shPARP2 C2C12 cells at *p* < 0.05, *p* < 0.01, and *p* < 0.001, respectively. ^##^ and ^###^ Statistically significant differences between the negative control and transiently transfected samples at *p* < 0.01 and *p* < 0.001, respectively.

**Figure 5:**
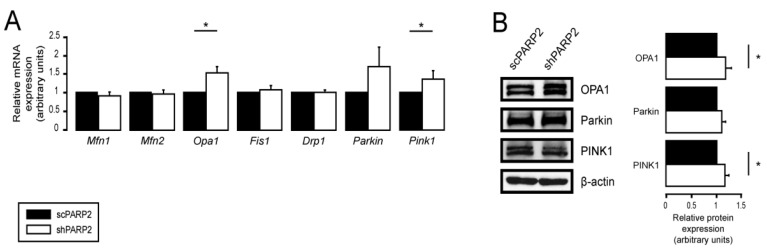
Deregulation of mitochondrial dynamics did not influence mitochondrial fragmentation. (**A**,**B**) A total of 200,000 scPARP2 or shPARP2 C2C12 cells were seeded into 6-well plates. (**A**) mRNA and (**B**) protein expression of the indicated mitochondrial fusion/fission genes were assessed by RT-qPCR (*n* = 4/6) or Western blotting (*n* = 3). Representative Western blot images are presented in (**B**). Numerical values are presented as the average ± SEM. Statistical significance was determined using paired, two-tailed Student’s *t*-test. * Statistically significant differences between the scPARP2 and shPARP2 C2C12 cells at *p* < 0.05.

Multiple parallel pathways can govern the fragmentation of the mitochondrial network. Therefore, we assessed these possibilities. The silencing of PARP2 blocks autophagy [58], which may affect mitophagy, leading to mitochondrial fragmentation. We assessed the co-localization of LC3, a marker of autophagic vesicles, and TOMM20, a mitochondrial marker; however, there was no overlap between the LC3+ (a marker for autophagosomes) and MitoTracker Red+ or TOMM20+ (both mitochondrial markers) features (Figure 4A). For a positive control demonstrating overlap of staining, C2C12 cells were co-stained with Tomm20 and MitoTracker Red (Supplementary Figure S1). Thus, PARP2 silencing did not increase mitophagy. SIRT1 activation is associated with PARP2 silencing [27,55,57], and SIRT1 regulates mitochondrial morphology [75,81]. However, genetic silencing of SIRT1 in shPARP2 cells did not reverse the changes to mitochondrial morphology (Figure 4B). We also checked the involvement of mtUPR, which was shown to play a role in mitochondrial biogenesis induced by pharmacological PARP inhibition [82]. However, no consistent induction in the expression of key heat shock proteins (Hsp25, Hsp40, Hsp60, Hsp70, Hsp90, Hsp110) were detected when scPARP2 and shPARP2 C2C12 cells were compared under control conditions or after heat shock (Supplementary Figure S2). These pathways are, therefore, unlikely to contribute to mitochondrial fragmentation. We observed a mild increase in the mRNA and protein expression of OPA1, Parkin, and Pink1 (Figure 5A,B).

### 3.3. Mitochondrial Fragmentation Is Not Specific for C2C12 Myoblasts

**Figure 6:**
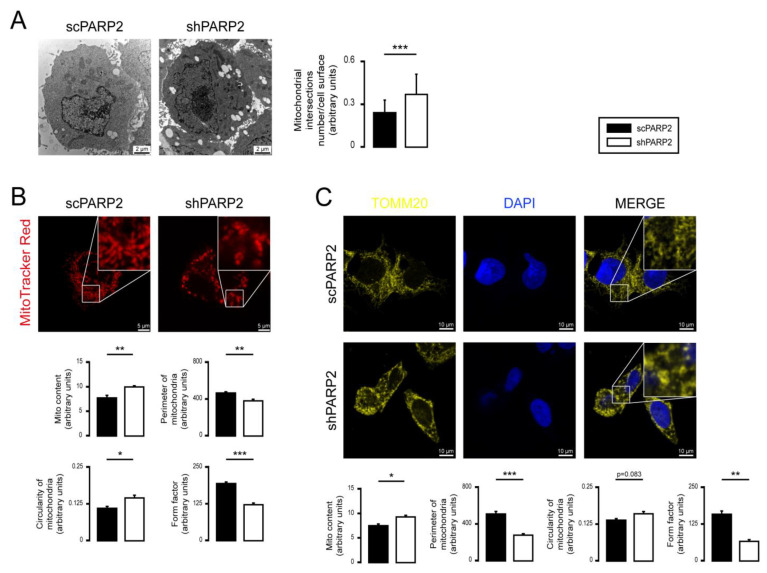
Silencing of PARP2 induces mitochondrial fragmentation in HepG2 cells. (**A**) Osmiated scPARP2 and shPARP2 HepG2 cells were analyzed by electron microscopy (*n* = 1; 45 cells were assessed from each group). Mitochondrial numbers were determined using ImageJ software, and mitochondrial intersections number/cell surface were plotted. (**B**,**C**) A total of 80,000 scPARP2 or shPARP2 HepG2 cells were seeded into 24-well plates on glass coverslips, and the cells were labeled with MitoTracker Red (*n* = 3) (**B**) or TOMM20 antibody (**C**) (*n* = 3). The nuclei were visualized using DAPI. Mitochondrial morphology was analyzed using ImageJ software with Mito-Morphology Macro (measured cells: 20/20). Representative Western blot and electron microscopic and immunofluorescence images are presented in the figure. Numerical values are presented as the average ± SEM in (**B**,**C**) and as the average ± SD in (**A**). Statistical significance was determined using paired, two-tailed Student’s *t*-test. *, **, and *** Statistically significant differences between the scPARP2 and shPARP2 HepG2 cells at *p* < 0.05, *p* < 0.01, and *p* < 0.001, respectively.

**Figure 7:**
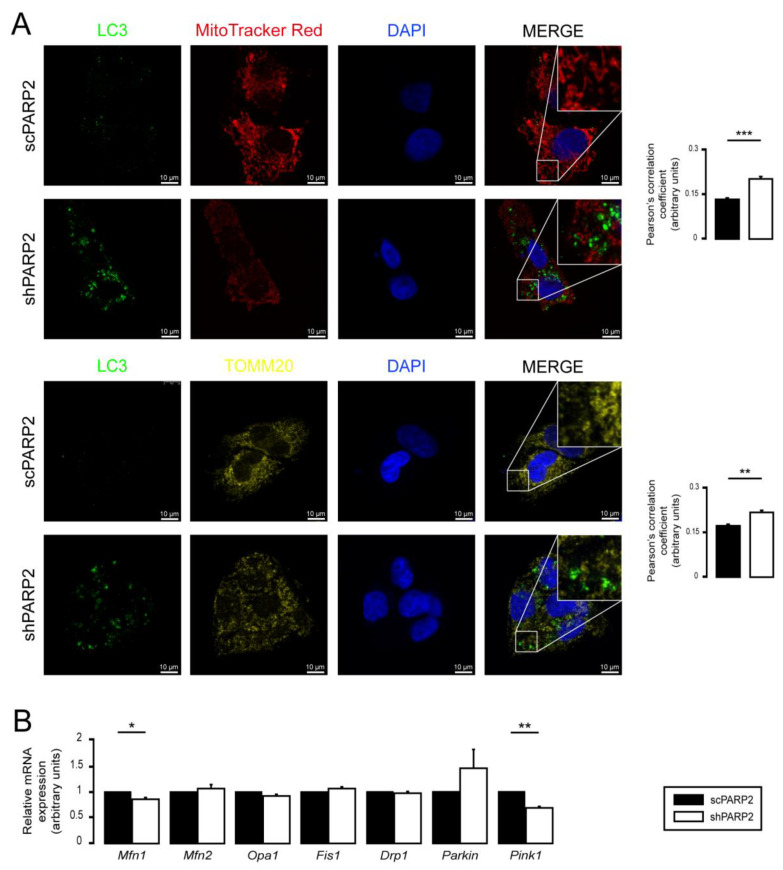
Mitophagy or deregulation of the mitochondrial fusion/fission system is not responsible for mitochondrial fragmentation in HepG2 cells. (**A**) A total of 80,000 scPARP2 or shPARP2 HepG2 cells were seeded into 24-well plates on glass coverslips, and LC3-MitoTracker Red–DAPI or LC3-TOMM20–DAPI co-immunofluorescence was performed (*n* = 3). Co-localization analysis was performed using ImageJ software with the EzColocalization plug-in (measured cells: 100/100). (**B**) A total of 250,000 scPARP2 or shPARP2 HepG2 cells were seeded into 6-well plates. The mRNA expression of mitochondrial fusion/fission genes was assessed by RT-qPCR (*n* = 3). Representative immunofluorescence images are presented in (**A**). Numerical values are presented as the average ± SEM. Statistical significance was determined using paired, two-tailed Student’s *t*-test. *, **, and *** Statistically significant differences between the scPARP2 and shPARP2 HepG2 cells at *p* < 0.05, *p* < 0.01, and *p* < 0.001, respectively.

We assessed another cell line in which PARP2 was silenced (scPARP2 and shPARP2 cell lines). Similar to shPARP2 C2C12 cells, in shPARP2 HepG2 cells also, we observed mitochondrial fragmentation marked by an increased number of mitochondrial cross sections on EM sections, decreased perimeter of individual mitochondria, decreased form factor, and increased circularity of mitochondria in MitoTracker-stained and TOMM20-stained cells (Figure 6A–C). Nevertheless, similar to C2C12 cells, no overlap between LC3- and TOMM20-positive features in shHepG2cells was detected (Figure 7A), suggesting that mitochondrial fragmentation is not due to increased mitophagy. We also assessed the mRNA expression of a set of genes involved in regulating mitochondrial morphology. The silencing of PARP2 decreased the expression of Mitofusin1 (Mfn1) and Pink1 (Figure 7B). The expression pattern of the genes involved in regulating mitochondrial morphology was not similar in shPARP2 C2C12 cells and shPARP2 HepG2 cells, making it unlikely that the deregulation of these genes could be responsible for mitochondrial fragmentation.

### 3.4. Silencing of PARP2 Leads to Oxidative Stress, Originating Partly from the Mitochondria through Mitochondrial Protein Imbalance and Fragmentation

**Figure 8:**
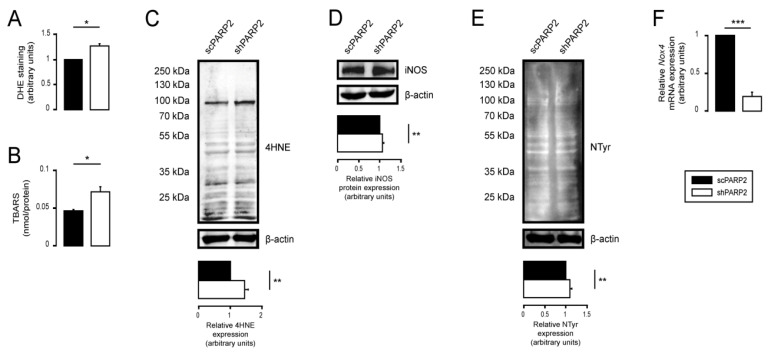
Oxidative stress is induced upon the silencing of PARP2. (**A**) A total of 200,000 scPARP2 or shPARP2 C2C12 cells were seeded into 6-well plates, and superoxide production was determined by dihydroethidium (DHE) staining (*n* = 3). (**B**) A total of 700,000 scPARP2 or shPARP2 C2C12 cells were seeded into T75 cell culture flasks, and lipid peroxidation was determined by TBARS assay (*n* = 5). Data were normalized to the cell number. (**C**–**E**) A total of 200,000 scPARP2 or shPARP2 C2C12 cells were seeded into a 6-well plate. 4-Hydroxynonenal (4HNE) (**C**), inducible nitric oxide synthase (iNOS) (**D**), or nitrotyrosine (NTyr) (**E**) expression was assessed by Western blotting (*n* = 3). (**F**) A total of 200,000 scPARP2 or shPARP2 C2C12 cells were seeded into 6-well plates, and the expression of Nox4 was determined by RT-qPCR (*n* = 3). Representative Western blot images are presented in (**C**–**E**). Numerical values are presented as the average ± SEM. Statistical significance was determined using paired, two-tailed Student’s *t*-test. *, **, and *** Statistically significant differences between the scPARP2 and shPARP2 C2C12 cells at *p* < 0.05, *p* < 0.01, and *p* < 0.001, respectively.

The redox state of cells changes in response to PARP inhibition (reviewed in [83,84]). The oxidative stress markers, dihydroethidine fluorescence, 4-hidroxynonenal (4HNE), and thiobarbituric acid-reactive substances (TBARS), increased in shPARP2 C2C12 cells (Figure 8A–C) compared with controls. Nitrosative stress markers did not exhibit large changes (Figure 8D–F).

**Figure 9:**
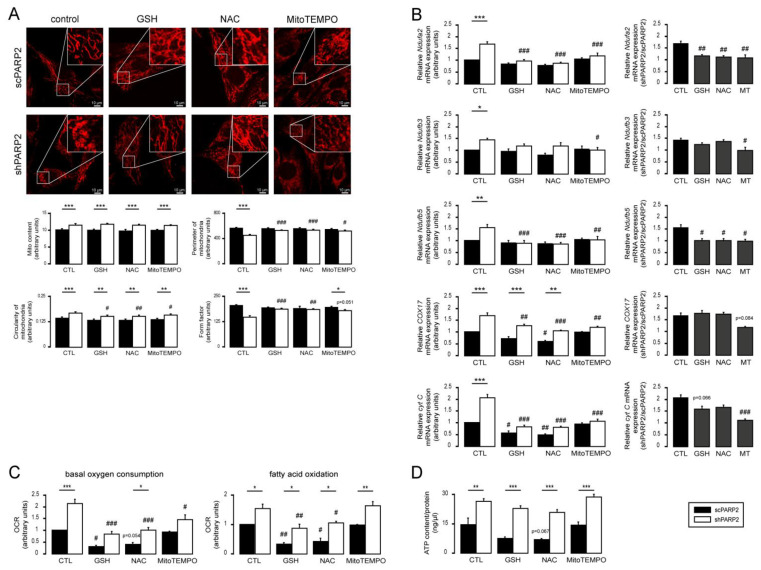
General reductants and MitoTEMPO can prevent mitochondrial fragmentation and mitochondrial biogenesis. (**A**) A total of 20,000 scPARP2 or shPARP2 C2C12 cells were seeded into 24-well plates on glass coverslips and treated with 5 mM glutathione (GSH), 5 mM N-acetyl-L-cysteine (NAC), or 10 µM MitoTEMPO for 48 h. After treatment, the mitochondrial structure was investigated using MitoTracker Red staining (*n* = 3). Mitochondrial morphology was analyzed using ImageJ software with Mito-Morphology Macro (measured cells: 20 from each group). (**B**) A total of 100,000 scPARP2 or shPARP2 C2C12 cells were seeded into 6-well plates and treated with 5 mM glutathione (GSH), 5 mM N-acetyl-L-cysteine (NAC), or 10 µM MitoTEMPO for 48 h. After treatment, mRNA expression of the indicated mitochondrial genes was assessed by RT-qPCR (*n* = 4). (**C**) A total of 2000 scPARP2 or shPARP2 C2C12 cells were seeded into 96-well assay plates and treated with 5 mM glutathione (GSH), 5 mM N-acetyl-L-cysteine (NAC), or 10 µM MitoTEMPO for 48 h. After treatment, Seahorse assays were performed in the presence or absence of etomoxir to assess total and fatty acid-dependent oxidation (*n* = 3). (**D**) A total of 100,000 scPARP2 or shPARP2 C2C12 cells were seeded into 6-well plates and treated with 5 mM glutathione (GSH), 5 mM N-acetyl-L-cysteine (NAC), or 10 µM MitoTEMPO for 48 h. ATP content was assessed using the ATP Assay Kit. Data were normalized to protein content, and normalized readings are displayed. Representative immunofluorescence images are presented in (**A**). Numerical values are presented as the average ± SEM. Statistical significance was determined using ANOVA followed by Tukey’s post hoc test, except for (**B**); in the shPARP2/scPARP ratio, Dunnett’s post hoc test was used. *, **, and *** Statistically significant differences between the scPARP2 and shPARP2 C2C12 cells at *p* < 0.05, *p* < 0.01, and *p* < 0.001, respectively. ^#^, ^##^, and ^###^ Statistically significant differences between control and treated samples at *p* < 0.05, *p* < 0.01, and *p* < 0.001, respectively.

To determine the effects of increased oxidative stress on mitochondrial fragmentation, we assessed how general reductants, such as reduced glutathione (GSH) or N-acetyl-cysteine (NAC), and a mitochondrial-targeted antioxidant MitoTEMPO affected mitochondrial morphology (similar to [69]). Although the antioxidants did not prevent mitochondrial biogenesis (marked by “Mito content” in Figure 9A), they affected the structure of the mitochondrial network (“Perimeter” and “Form factor” in Figure 9A). All three antioxidants increased the perimeters and form factors in mitochondria and slightly decreased the circularity of mitochondria in shPARP2 cells (Figure 9A). These results suggest that mitochondrial fragmentation relies on oxidative stress of mitochondrial origin.

**Figure 10:**
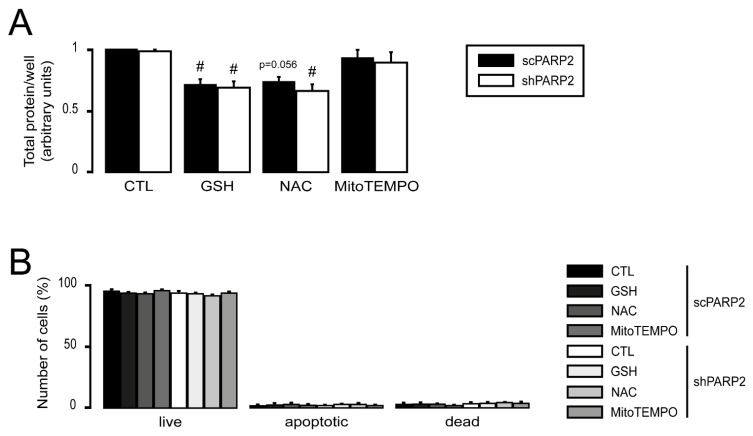
Silencing of PARP2 does not affect cell proliferation or spontaneous cell death. (**A**) A total of 2000 scPARP2 or shPARP2 C2C12 cells were seeded into 96-well plates and treated with 5 mM glutathione (GSH), 5 mM N-acetyl-L-cysteine (NAC), or 10 µM MitoTEMPO for 48 h. After treatment, cell proliferation (SRB) assays were performed (*n* = 3). (**B**) A total of 100,000 scPARP2 or shPARP2 C2C12 cells were seeded into 6-well plates and treated with 5 mM glutathione (GSH), 5 mM N-acetyl-L-cysteine (NAC), or 10 µM MitoTEMPO for 48 h. Cell death was assessed using the FITC Annexin V/Dead Cell Apoptosis Kit. Numerical values are presented as the average ± SEM. Statistical significance was determined using ANOVA followed by Tukey’s post hoc test. ^#^ Statistically significant differences between control and treated samples at *p* < 0.05.

We assessed how the expression of mitochondrial genes responds to antioxidant treatment. The expression of Ndufa2, Ndufb3, Ndufb5, Cox17, and cyt C was induced in shPARP2 C2C12 cells compared with scPARP2 cells (Figure 9B). The expression of all genes reduced upon treatment with GSH, NAC, and MitoTEMPO (Figure 9B), similar to [69]. These responses were translated into physiological changes, as the antioxidants reduced mitochondrial oxygen consumption (Figure 9C), fatty acid oxidation (Figure 9C), and ATP content (Figure 9D). There were no differences in cell proliferation and spontaneous cell death between scPARP2 and shPARP2 cells, even in the presence of GSH, NAC, and MitoTEMPO (Figure 10A,B).

### 3.5. PARP1 and PARP3 Are Not Involved in Mitochondrial Fragmentation Elicited by the Silencing of PARP2

**Figure 11:**
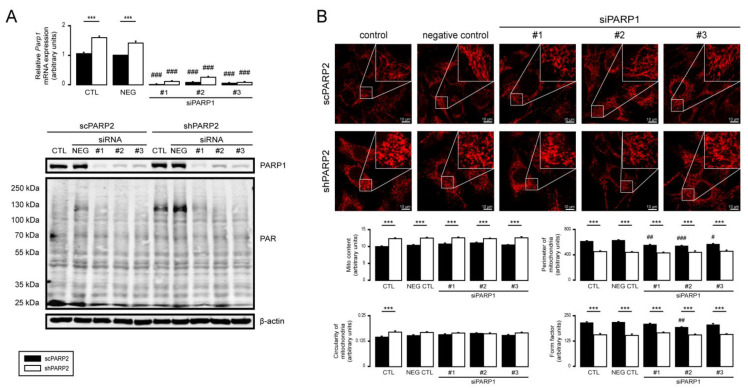
Silencing of PARP1 does not influence the mitochondrial morphology induced by the silencing of PARP2. (**A**,**B**) A total of 30,000 scPARP2 or shPARP2 C2C12 cells were seeded into 24-well plates, and PARP1 was transiently silenced using three types of siRNAs (*n* = 3). (**A**) Cells were assessed 48 h post-transfection, and then the expression of PARP1 was determined using RT-qPCR (*n* = 3). Expression of PARP1 and PAR was determined using Western blotting (*n* = 3). (**B**) In the same cells, the mitochondrial structure was investigated using MitoTracker Red staining (*n* = 3). Mitochondrial morphology was analyzed using ImageJ software with Mito-Morphology Macro (measured cells: 20 from each group). Representative Western blot and immunofluorescence images are presented in the figure. Numerical values are presented as the average ± SEM. Statistical significance was determined using ANOVA followed by Tukey’s post hoc test. *** Statistically significant differences between the scPARP2 and shPARP2 C2C12 cells at *p* < 0.05. ^#^, ^##^, and ^###^ Statistically significant differences between the negative control and transiently transfected samples at *p* < 0.05, *p* < 0.01, and *p* < 0.001, respectively.

**Figure 12:**
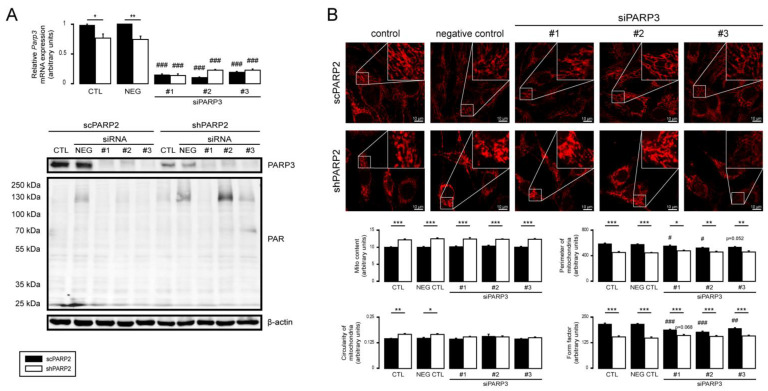
Silencing of PARP3 does not influence the mitochondrial morphology induced by the silencing of PARP2. (**A**,**B**) A total of 30,000 scPARP2 or shPARP2 C2C12 cells were seeded into 24-well plates, and PARP3 was transiently silenced using three types of siRNAs (*n* = 3). (**A**) Cells were assessed 48 h post-transfection, and then the expression of PARP3 was determined using RT-qPCR (*n* = 3). Expression of PARP3 and PAR was determined using Western blotting (*n* = 3). (**B**) In the same cells, the mitochondrial structure was investigated using MitoTracker Red staining (*n* = 3). Mitochondrial morphology was analyzed using ImageJ software with Mito-Morphology Macro (measured cells: 20 from each group). Representative Western blot and immunofluorescence images are presented in the figure. Numerical values are presented as the average ± SEM. Statistical significance was determined using ANOVA followed by Tukey’s post hoc test. *, **, and *** Statistically significant differences between the scPARP2 and shPARP2 C2C12 cells at *p* < 0.05, *p* < 0.01, and *p* < 0.001, respectively. ^#^, ^##^, and ^###^ Statistically significant differences between the negative control and transiently transfected samples at *p* < 0.05, *p* < 0.01, and *p* < 0.001, respectively.

PARP1 and PARP3 were implicated in regulating mitochondrial morphology [20,85,86]; hence, we assessed the possible involvement of PARP1 and PARP3. Silencing PARP1 in scPARP2 and shPARP2 C2C12 cells (Figure 11A) led to the loss of PARP1 auto-PARylation (Figure 11A). Silencing of PARP1 induced mild mitochondrial fragmentation in scPARP2 cells; nevertheless, it did not influence the morphology in shPARP2 cells (Figure 11B), suggesting little or no involvement in the mitochondrial phenotype in shPARP2 cells. When PARP3 was silenced (Figure 12A), upon the application of the negative control, unspecific siRNA, and two specific siRNA, activation of PARP1 was observed (Figure 12A). Similar to the case of PARP1, silencing of PARP3 in scPARP2 C2C12 cells induces mild mitochondrial fragmentation but does not change the phenotype of the shPARP2 C2C12 cells (Figure 12B), suggesting little or no involvement in the mitochondrial phenotype in shPARP2 cells.

## 4. Discussion

Previous studies have shown that the genetic deletion of PARP1 or PARP2 [55,87,88,89,90,91,92,93] or pharmacological PARP inhibition [82,87,94] induces mitochondrial biogenesis in multiple organs and tissues, including skeletal muscle. In this study, we provide evidence for fragmentation of the mitochondrial network via induction of oxidative stress in cells. Reduced glutathione (GSH) and N-acetyl-cysteine (NAC), which are known antioxidants that minimize the effect of oxidative stress, reversed the changes to mitochondrial morphology that occur when PARP2 expression is decreased. The mitochondria-targeted antioxidant MitoTEMPO also affected the structure of the mitochondrial network, suggesting that the fragmentation of the mitochondrial network leans on the oxidative stress of mitochondria. GSH, NAC, and MitoTEMPO also reduced mitochondrial oxidative phosphorylation. In good agreement with these results, pharmacological PARP inhibition by olaparib or nicotinamide, as well as increased cellular NAD^+^ levels [75,76,77,78,79,80], also induce mitochondrial fragmentation [20]. PARP1 or PARP3 does not compensate for the loss of PARP2 in C2C12 cells. Furthermore, the silencing of PARP1 or PARP3 can bring about signs of mitochondrial fragmentation.

Although SIRT1 activation in the absence of PARP2 was shown to induce mitochondrial biogenesis [27,55,57] and SIRT1 can regulate mitochondrial morphology [75,81], modulating SIRT1 activity does not influence mitochondrial fragmentation. Silencing of PARP2 induces autophagy [58], making it likely that mitochondrial fragmentation is due to enhanced mitophagy. However, we ruled out the involvement of mitophagy, despite the indication that PARP1 inhibition can induce mitophagy [18]. Furthermore, in the absence of PARP2, the consistent dysregulation of the mitochondrial fusion/fission machinery and the induction of mtUPR were both ruled out.

Treatment of cells with GSH, NAC, or MitoTEMPO reduced the expression of mitochondrial genes and reduced mitochondrial output but did not reduce mitochondrial content in shPARP2 cells. Taken together, mitochondrial biogenesis upon the absence of PARP2 is dependent on the combined action of increased expression and NAD^+^-dependent activation of SIRT1 and mitochondrial reactive species production. SIRT1 activation [20,82,86] and reactive species production [95,96,97] were linked with mitonuclear proteostasis in other models. Yet, the source of reactive species has not been identified. Nevertheless, higher mitochondrial activity in a cell leads to higher levels of oxygen-centered reactive species in the Szent–Györgyi–Krebs cycle and the mitochondrial electron transport chain [98].

PARP2 has physiological roles in skeletal muscle. Namely, the silencing of PARP2 supports isotype switching, inducing proportions of slow-twitch, type I fibers and supporting mitochondrial biogenesis through inducing SIRT1 [55,57]. Furthermore, the silencing of PARP2 induces the expression of myogenic transcription factors in differentiating C2C12 myoblasts [58], suggesting pro-differentiation effects, while at the same time, the silencing of PARP2 leads to an aberrant stress fiber-like actin structure [58]. The role of PARP2 in skeletal muscle biology warrants further examination. Nevertheless, there is a large set of human diseases involving PARP activation and mitochondrial damage [53,99,100,101,102,103]; hence, our results, in terms of mitochondrial output and mitochondrial morphology, have implications for understanding the protective action of PARP inhibitors in these diseases.

## Figures and Tables

**Table 1:** List of antibodies used in immunofluorescence.

Antibody	Company	Catalog Number	Dilution
TOMM20	Abcam (Cambridge, MA, USA)	ab56783	1:200
LC3A/B Alexa Fluor 488 conjugate	Cell Signaling Technology (Danvers, MA, USA)	13082	1:50
Goat-anti mouse IgG Alexa Fluor 546	Thermo Fisher Scientific (Walthan, MA, USA)	A-11003	1:1000

TOMM20: translocase of outer mitochondrial membrane 20.

**Table 2:** List of RT-qPCR primers used in the study.

Gene Name	Primers
COX17	5′-CGTGATGCGTGCATCATTGA-3′ 5′-CATTCACAAAGTAGGCCACC-3′
Cyclophilin	5′-TGGAGAGCACCAAGACAGACA-3′ 5′-TGCCGGAGTCGACAATGAT-3′
Cyt C	5′-TCCATCAGGGTATCCTCTCC-3′ 5′-GGAGGCAAGCATAAGACTGG-3′
Drp1	5′-TCGAGTCCCCATTCATTGCAGT-3′ 5′-GAAGAAGGTCCCTGCCCACTAG-3′
Fis1	5′-CTGGACTCATTGGACTGGCTGT-3′ 5′-AGAGGTAGACTACAGGGGTGCA-3′
Mfn1	5′-GGTCCTGCAATCACTCTGTCCT-3′ 5′-CCCATTTCACCCCTTCAGACCT-3′
Mfn2	5′-GTGATCAGGTTCAGCGTCCTCT-3′ 5′-CCACTCCTCCGACCACAAGAAT-3′
Ndufa2	5′-GCACACATTTCCCCACACTG-3′ 5′-CCCAACCTGCCCATTCTGAT-3′
Ndufb3	5′-TACCACAAACGCAGCAAACC-3′ 5′-AAGGGACGCCATTAGAAACG-3′
Ndufb5	5′-CTTCGAACTTCCTGCTCCTT-3′ 5′-GGCCCTGAAAAGAACTACG-3′
Nox4	5′-GCAGATTTACTCTGTGTGTTGCAT-3′ 5′-TCCCATCTGTTTGACTGAGGT-3′
Opa1	5′-ATTGTGTGCTCTCAGTCAGGCT-3′ 5′-ACCTTTCCCTGACGCCTAGTTC-3′
Parkin	5′-AAATGCATCTGGAGGGGACGAA-3′ 5′-TAACTGGACCTCTGGCTGCTTC-3′
Parp1	5′-GGAGCTGCTCATCTTCAACC-3′ 5′-GCAGTGACATCCCCAGTACA-3′
Parp2	5′-GGAAGGCGAGTGCTAAATGAA-3′ 5′-AAGGTCTTCACAGAGTCTCGATTG-3′
Parp3	5′-CCTGCTGATAATCGGGTCAT-3′ 5′-TTGTTGTTGTTGCCGATGTT-3′
Pink1	5′-GGGAAGAACAGCCTTGAACAGC-3′ 5′-GCAAAGTTCAGTGTTGGCCTCA-3′
Sirt1	5′-TGTGAAGTTACTGCAGGAGTGTAAA-3′ 5′-GCATAGATACCGTCTCTTGATCTGAA-3′
36B4	5′-AGATTCGGGATATGCTGTTGG-3′ 5′-AAAGCCTGGAAGAAGGAGGTC-3′

Ndufa2/Ndufb3/Ndufb5: NADH:ubiquinone oxidoreductase subunit A2/B3/B5; Cox17: cytochrome c oxidase; Cyt C: cytochrome c; Sirt1: sirtuin 1; Mfn1: mitofusin 1; Mfn2: mitofusin 2; Opa1: optic atrophy 1; Fis1: fission; Drp1: dynamin-1-like protein; Pink1: PTEN induced kinase 1; PARP1: poly(ADP-ribose) polymerase-1, PARP3: poly(ADP-ribose) polymerase-3, Nox4: NADPH oxidase 4.

**Table 3:** List of antibodies used for Western blotting.

Antibody	Company	Catalog Number	Dilution
GAPDH	Sigma-Aldrich	G9545	1:10,000
HSP25	Enzo Life Sciences	ADI-SPA-801	1:1500
HSP70/HSP72 (C92F3A-5)	Enzo Life Sciences	ADI-SPA-810	1:8000
HSC70/HSP70 (N27F3-4)	Enzo Life Sciences	ADI-SPA-820	1:1000
HSP40 (C-20)	Santa Cruz Biotechnology	sc-1800	1:1000
HSP90 (AC88)	Enzo Life Sciences	ADI-SPA-830	1:1000
HSP110	Becton Dickinson Biosciences	610510	1:2500
iNOS	Novus Biologicals	NB300-605	1:1000
NTyr	Millipore	06-284	1:1000
OPA1	Thermo Fisher Scientific	MA5-16149	1:1000
Parkin	Thermo Fisher Scientific	PA5-13399	1:1000
PARP1	Cell Signaling Technology	9532	1:2000
PARP2	Enzo Life Sciences	ALX-210-899	1:2000
PARP3	Novus Biologicals	NBP1-31415	1:1000
PAR	Sigma-Aldrich	MABC547	1:1000
PINK1	Novus Biologicals	BC100-494	1:1000
SIRT1	Millipore	07-131	1:1000
4HNE	Abcam	ab46545	1:1000
Anti-mouse IgG, HRP-linked	Sigma-Aldrich	A9044	1:2000
Anti-rabbit IgG, HRP-linked	Cell Signaling Technology	7074	1:2000
Anti-β-actin-peroxidase	Sigma-Aldrich	A3854	1:20,000

HNE: 4-hydroxynonenal, iNOS: inducible nitric oxide synthase; NTyr: nitrotyrosine; HSP: heat shock protein; HSC: heat shock cognate protein; GAPDH: glyceraldehyde-3-phosphate dehydrogenase.

## Data Availability

All primary data are uploaded to FIGSHARE at https://figshare.com/s/afe02e5a75b4a1bd584c (doi:10.6084/m9.figshare.12570068).

## Supplementary Materials

The following are available online at https://www.mdpi.com/article/10.3390/cells10061387/s1, Figure S1: Mitochondrial fragmentation in C2C12 is not due to mitophagy or SIRT1 activation, Figure S2: Lack of consistent difference in HSP expression and induction in scPARP2 andshPARP2 cells.
