# Supplementary material for: Silencing of Poly(ADP-Ribose) Polymerase-2 Induces Mitochondrial Reactive Species Production and Mitochondrial Fragmentation
Source: Cells. 2021 Jun 4;10(6):1387. doi: 10.3390/cells10061387 (PMC8227884; doi:10.3390/cells10061387)
Supplement: Supplementary file 1 [file cells-10-01387-s001.zip › cells-1103272-supplementary.pdf]

Supplementary information for Jankó et al. Silencing of poly(ADP-ribose) polymerase-2 induces mitochondrial reactive species production and mitochondrial fragmentation

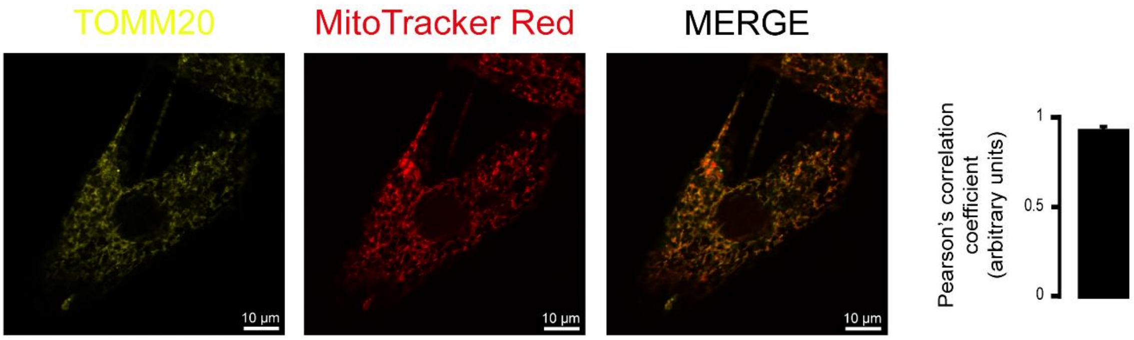

Supplementary Figure 1. Mitochondrial fragmentation in C2C12 is not due to mitophagy or SIRT1 activation. A total of 70,000 C2C12 cells were seeded into 24-well plates on glass coverslips and LC3-MitoTracker Red and LC3-TOMM20 co-immunofluorescence was performed (n=3). Colocalization analysis was performed using ImageJ software with the EzColocalization plugin (measured cells: 100/100). Representative immunofluorescence images are presented in the figure. Numerical values are presented as the average ± SD.

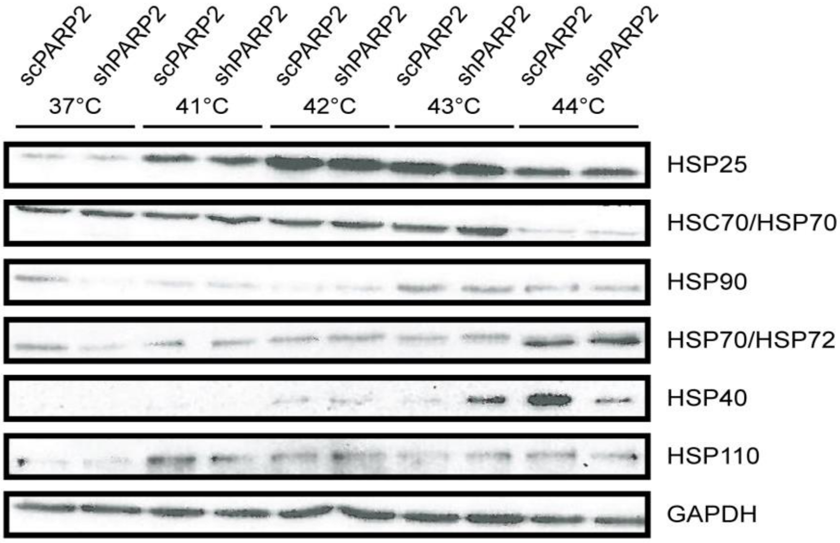

Supplementary Figure 2. Lack of consistent difference in HSP expression and induction in scPARP2 and shPARP2 cells. scPARP2 and shPARP2 cells (n=70 000 cells/sample) were seeded and were kept at 37 oC or were subjected to heat shock as indicated for 1 hour, followed by 16 hours of recovery. Cells were harvested and were cellular proteins were subjected to SDS-PAGE and Western blotting with the antibodies indicated. Sample blots are provided.
